# Adjustment Disorder in the Face of COVID-19 Outbreak: The Impact of Death Anxiety, Media Exposure, Fear of Contagion and Hypochondriasis Symptoms

**DOI:** 10.1177/00302228211034372

**Published:** 2021-07-29

**Authors:** Michal Mahat-Shamir, Shani Pitcho-Prelorentzos, Maya Kagan, Miri Kestler-Peleg, Osnat Lavenda

**Affiliations:** School of Social Work, Ariel University, Israel

**Keywords:** adults and death, death anxiety, coping/adaptation, disaster, disease

## Abstract

Based on the theoretical view of Terror Management Theory, the current research examines whether higher levels of death anxiety symptoms, in the face of the COVID-19 outbreak, increase the extent to which participants are exposed to information regarding the spread of the pandemic, as well as the fear of contagion and symptoms of hypochondriasis, which all in turn increase symptoms of adjustment disorder. A total number of 302 participants filled out self-report questionnaires regarding death anxiety, adjustment disorder, the extent of exposure to information regarding COVID-19, fear of contagion, hypochondriasis, and demographic information. Structural Equation Modeling analysis indicated a very good fit of the theoretical model with the data, confirming the mediation effect of exposure to information, fear of contagion, and symptoms of hypochondriasis on the association between death anxiety and adjustment disorder symptoms. Implications for practice are discussed.

In December 2019, Wuhan and gradually other places of China have experienced an outbreak of pneumonia epidemic caused by the 2019 novel coronavirus (COVID-19) (Zhang et al., 2020). The spread of COVID-19 has taken on pandemic proportions, affecting over 100 countries in a matter of weeks (Remuzzi & Remuzzi, 2020), including Israel. Thus, the World Health Organization (WHO) has declared the COVID-19 a public health emergency of international concern (WHO, 2020). As the world is increasingly becoming a “global village,” virtually everyone in Israel were exposed to images of the COVID-19 outbreak through the media, thus to a related threat: psychological distress resulting from exposure to the outbreak. Indeed, heightened stress responses during and in the immediate aftermath of a threatening event are associated with adverse physical and mental health outcomes over time (Garfin et al., 2020). One of these mental health outcomes may be adjustment disorder.

Adjustment disorder is a maladaptive reaction to a stressful event, to ongoing psychosocial difficulties or to a combination of stressful life situations that usually emerges within a month of the occurrence of a stressor and tends to resolve within 6 months, unless the stressor persists for a longer duration (Maercker et al., 2013). The reaction to the stressor is characterized by symptoms of preoccupation with the stressor, such as excessive worry, recurrent and distressing thoughts about the stressor or constant rumination about its implications. There is a failure to adapt, which is manifested in symptoms interfering with everyday functioning, such as difficulties concentrating or sleep disturbances. The symptoms can also be associated with loss of interest in work, social life, caring for others, and leisure activities, resulting in impairment in social or occupational functioning (restriction of social network, conflicts in family, absenteeism and so on) (Maercker et al., 2013).

The COVID-19 pandemic may serve as a stressful health event that triggers a maladaptive reaction in the form of adjustment disorder. The current research seeks to explore the underlying mechanisms that may trigger this maladaptive reaction. Based on the theoretical view of terror management theory (TMT; Greenberg et al., 1997), we assume that the main trigger for adjustment disorder symptoms is the heightened levels of death anxiety that individuals may experience in the face of the life-threatening COVID-19 pandemic. Additionally, we assume that death anxiety symptoms increase the extent to which individuals are exposed to information regarding the spread of the pandemic, as well as the fear of contagion and symptoms of hypochondriasis, which all in turn increase symptoms of adjustment disorder.

## Death Anxiety

A threat to one's health and death anxiety are not only logically related (i.e., illness may lead to death), but also appear to be driven by core, underlying emotion regulation mechanisms. In this regard, one of the most influential theories which explores the manner by which individuals deal with exposure to death-related stimuli is TMT (Greenberg et al., 1997) and its extension: anxiety buffer disruption theory (Pyszczynski & Kesebir, 2011). One of the main theoretical postulations of TMT is that as humans we are aware of our own mortality; this knowledge creates extreme levels of stress and anxiety. The inevitability of death, as well as our perceived lack of control over its circumstances, creates an internal sense of anxiety, which makes life extremely difficult. In other words, the juxtaposition of death awareness, presumably a uniquely human capacity made possible by cognitive abilities such as self-awareness and abstract thought, and the instinct for self-preservation, common to all animals, creates a challenging and harsh living situation.

TMT holds that when faced with the reality of their own mortality, individuals cope with the anxiety (or ‘terror’) of this by drawing on sources of meaning that connect them to aspects of their selves or lives that will live on after they die (Solomon et al., 1991). The awareness of one’s personal mortality is known as the mortality salience effect. Three common buffers that minimize the anxiety of mortality salience include affirmation of (a) one’s cultural worldview, (b) the self and one’s personal values, and (c) one’s significance in the context of close personal relationships. When thoughts of death are evoked and individuals are not able to utilize these psychological buffers, otherwise well-functioning individuals, show lower satisfaction with life, less subjective vitality, poorer meaning in life, greater negative affect, a reduced tendency to explore and attempt new and challenging tasks, and greater anxiety (Routledge et al., 2010).

A study previously conducted in Israel indicated that increased concerns over one’s security situation are connected with enhanced death anxiety (Mahat-Shamir et al., 2018). This previous study was conducted in relation to ongoing terror attacks, however, empirical studies in the field of TMT have illustrated that when individuals do not have the capacity to ‘defend’ against the threat of illness, such as by actively suppressing thoughts related to illness or death, activating the threat of illness activates the threat of death (Arndt et al., 2007). Low ability to suppress threat related thoughts may lead to an extensive exposure to that threat. We assume the threat of death and dying, posed by the COVID-19 pandemic, may serve as a stressful life situation. The stress evoked by the pandemic may trigger illness-related worries and therefore, death awareness and death anxiety. The inevitability of death, as well as the perceived lack of control over its circumstances, may lead individuals to seek control through frequent exposure to information regarding the spread of the pandemic. Moreover, death anxiety may affect one’s cognitions and behaviors, that will manifest according to the nature of the stressor (i.e., health-related threat) such as, heighten fear of contagion and increase hypochondriasis symptoms, which may all lead to a maladaptive reaction in the form of adjustment disorder.

## Exposure to Information

Studies indicate the significance of physical proximity to the site of a stressful event and its diverse impact on the extent to which individuals develop stress-related disorders (Grieger et al., 2005). As tens of thousands of people are affected by traumatic or stressful events directly, many more are affected indirectly; namely, family members, friends, rescue workers, or residents living in the vicinity of the event (S. C. Thompson et al., 2006). An indirect key factor in the spread of the effects of stressful events is the mass media, which act as conduits in affecting the general public. Indeed, correlations were found between media exposure to stressful events and anxiety symptoms (Cohen et al., 2006).

Heightened levels of death anxiety may lead individuals to seek more information regarding the life-threatening situation in an attempt to gain some control over its circumstances, especially in times of a global health crisis (Tausczik et al., 2012). Thus, the exposure to stressful situations is, in many cases a voluntary act, frequently self-selected. People usually decide to watch (or not) TV news and special reports on stressful events, or to seek specific information about it on the Internet (Garfin et al., 2020). Nevertheless, exposure to stressful events may also occur involuntarily when overhearing a conversation, changing TV channels randomly or searching the Internet for other information (Ben-Zur et al., 2012). Both news media and social media often provide a broad and detailed coverage of such stressful events, producing the threat of becoming aware of one’s own vulnerability and mortality as well as the possibility of experiencing anxiety and stress-related disorders (Ring et al., 2018; Tausczik et al., 2012). Importantly, involuntarily exposure may also be the result of talking to other people such as family members and friends. Thus, the reality is that virtually everyone is exposed to some information of stressful events through the media, especially in extremely stressful times, such as those of a worldwide pandemic outbreak. Indeed, in Israel very detailed information about COVID-19 was provided in the public media (Schiff et al., 2020). These public fears may escalate, fuel rumors, and provoke stress responses (Garfin et al., 2020), such as adjustment disorder.

## Fear of Contagion

Information containing practical advice on how to protect oneself from contagious viruses may be beneficial, and may also prevent other common contagions (e.g., influenza) (Hong & Collins, 2006). Nevertheless, heightened mortality salience due to an outbreak of a life-threatening pandemic, may also heighten perceived risk and fear about health-related topics (Ng et al., 2018; Wang et al., 2019), such as fear of contagion.

Fear of contagion was evident in Israel during the COVID-19 pandemic outbreak as for example, the increase in the number of COVID-19 inpatients was accompanied by a decrease in the number of emergency room admissions, the latter from about 1,40,000 each year during 2016–2019 to 1,11,750 during 2020. This was explained by the reluctance of patients with other acute conditions or exacerbations of chronic medical conditions to approach the emergency room for fear of contagion (Schreiber et al., 2021).

The correlation between fear of contagion and death anxiety was claimed by Cohen (1989) who described a form of HIV discrimination tailed “AIDSism.” In subsequent articles (Cohen & Alfonso, 1994) “AIDSism” is described as built on a foundation of homophobia, addictophobia, misogyny, fear of contagion, and fear of death. As the two constructs are correlated, it may be assumed that higher the levels of death anxiety are, the higher the levels of fear of contagion with a life-threatening disease such as COVID-19. Subsequently, fear of contagion may evoke maladaptive symptoms such as preoccupation with the stressor, excessive worry, recurrent and distressing thoughts about the stressor or constant rumination about its implications – all of which characterize adjustment disorder. As mentioned above, death anxiety symptoms may affect one’s cognitions and behaviors, that will manifest according to the nature of the stressor (i.e., health-related threat). Therefore, possible effects may be a heightened fear of contagion and increased hypochondriasis symptoms, which we will now elaborate on.

## Hypochondriasis Symptoms

Hypochondriasis is an excessive fear of, or preoccupation with, a serious illness. Medically unexplained symptoms are persistent bodily complaints for which adequate examination does not reveal sufficient explanatory structural or other specified pathology (Olde Hartman et al., 2004). Both, hypochondriasis and medically unexplained symptoms, are common conditions in the general population, but only in case of significant distress or impairment in functioning, these conditions are considered psychiatric disorders. In DSM-V (American Psychiatric Association [APA], 2013) these conditions were classified within the section of somatoform disorders, under somatic symptom disorder (SSD). This classification emphasizes the unexplained physical symptoms salience. More specifically, somatic symptom disorder (SSD) involves the manifestation of dysfunctional cognitions, emotions or behavior in relation to physical symptoms (APA, 2013). In DSM-V, the concept of hypochondriasis is partly reflected in illness anxiety disorder and partly in somatic symptom disorder, two separate disorders within the grouping of somatic symptom and related disorders (formerly known as somatoform disorders) (APA, 2013). The common feature across different disorders within the somatic symptom and related disorders grouping is the prominence of somatic symptoms associated with significant distress and impairment. This grouping comprises somatic symptom disorder, illness anxiety disorder, conversion disorder, psychological factors affecting other medical conditions, and factitious disorders. Depending on the symptoms of the individual, and particularly on the presence or absence of somatic symptoms, an individual patient that would have been diagnosed with hypochondriasis in DSM-IV will be diagnosed in DSM-V as having an illness anxiety disorder (if the somatic symptoms are absent or mild) or as having somatic symptom disorder (if one or more somatic symptoms are present and are distressing or result in significant disruption of daily life) (van den Heuvel et al., 2014).

Death anxiety can be a basic fear underlying the development, course, and maintenance of hypochondriasis (Arndt et al., 2005; Furer & Walker, 2008) through several mechanisms which may be considered about how death anxiety might contribute to developing or maintaining hypochondriasis. This ranges from fear of bodily failure with consequently somatic amplification, to death anxiety as an expression of separation fear (Aan de Stegge et al., 2018). As research indicate that mortality salience impact health-related behaviors (Goldenberg & Arndt, 2008), during a health crisis, such as in the case of the COVID-19 pandemic, high levels of death anxiety may trigger hypochondriasis symptoms. These maladaptive symptoms may lead to adjustment disorder symptoms such as preoccupation with the stressor, excessive worry, recurrent and distressing thoughts about the stressor or constant rumination about its implications as well as to an actual interference with everyday functioning.

**Figure 1.**
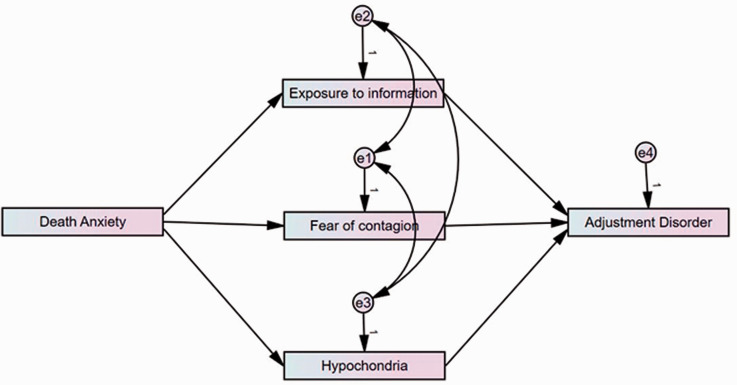
Results of the Mediation Model.

According to all of the above, in the current research we wished to examine whether higher levels of death anxiety symptoms, in the face of the COVID-19 outbreak, increase the extent to which participants are exposed to information regarding the spread of the pandemic, as well as the fear of contagion and symptoms of hypochondriasis, which all in turn increase symptoms of adjustment disorder. Thus, we hypothesized that exposure to information, fear of contagion and hypochondriasis symptoms will mediate the association between death anxiety and adjustment disorder (see Figure 1).

## Method

### Participants and Procedure

The sample of the present study consisted of 302 Israelis recruited through social media, between March 4th and March 15th, 2020, a time when there were less than 220 confirmed cases of COVID-19 in Israel and no deaths (Ministry of Health, Israel, 2020). Participants’ age ranged between 18 and 79 years old (*M* = 36.7, *SD* = 12.5), most of them were women (84.8%) and more than half (58.3%) reported being in a committed relationship. Most of the sample (83.1%) reported having an academic degree, about half (53.3%) reported working full time, and another quarter (25.8%) reported working part time.

After receiving the approval of the ethics committee at the authors’ University, a link to an electronic questionnaire was disseminated through social media groups (such as Facebook, WhatsApp etc.). The participants were asked to sign an electronic informed consent and respond to a questionnaire dealing with the psychological impacts of the pandemic. The questionnaire included screening measures for death anxiety, adjustment disorder and hypochondriasis as well as information regarding the extent to which participants were exposed to information regarding the spread of coronavirus, participants’ fear of coronavirus contagion and their demographic information. The questionnaire was anonymous and participants were not offered a fee or any price for their participation.

### Measures

In addition to information regarding socio-demographic characteristics, participants responded to the following measures:

***Death anxiety symptoms*** were measured using the Death Anxiety Questionnaire (DAQ) developed by Conte et al. (1982). This scale was used throughout the years with various populations, including Israeli populations (Hamama-Raz et al., 2016; Mahat-Shamir et al., 2018), and was proved to be valid across cultures (Jong et al., 2019). Cronbach’s alpha calculated in the present study was 0.870. Participants were asked to indicate the extent to which they experience each of the 15 scale statements on a three-point scale from 1 ‘Not at all’, through 2 ‘To some extent’ to 3 ‘Very much’.

***Hypochondriasis symptoms*** were measured using a 14-item scale developed by Pilowsky (1967). The scale measures three dimensions of hypochondriasis: “bodily preoccupation”, “disease phobia”, and “conviction of presence of disease with non-response to reassurance”. Its use is widely accepted across cultures (McDowell, 2006) and was validated on Israeli population (Mishali et al., 2020). Cronbach’s alpha calculated in the present study was 0.911. Participants were asked to rate the extent to which each statement describes their health, on a five-point Likert scale from 1 ‘Not at all’ to 5 ‘extremely’.

***Fear of contagion*** was measured by a single item developed for the purpose of the present study asking the participants “How much do you fear of getting coronavirus disease?”. Participants were asked to rate their fear on a scale with five degrees, from 1 ‘Not at all’ to 5 ‘Extremely’.

***Exposure to information regarding the outbreak of coronavirus disease*** was measured by a single item developed for the purpose of the present study asking participants “What is the frequency in which you receive information regarding the outbreak of coronavirus disease?”. Participants were asked to rate the frequency on a scale with five degrees, from 1 ‘Not at all’ to 5 ‘Several times a day’.

***Adjustment disorder symptoms*** were measured using the Ultra-brief-ADNM-4, a measure that is based on the Adjustment Disorder New Module (Ben-Ezra et al., 2018). The 20-item scale is widely used worldwide, and its validation was well established (Ben-Ezra et al., 2020; Lorenz et al., 2016). Its shortened version was validated and revalidated with Israeli samples and recently with Chinese sample (Liang et al., 2021). Cronbach’s alpha calculated for the present study was 0.868. Participants were asked to indicate the frequency of items on a 4-point Likert scale from 1 ‘never’ to 4 ‘often’.

### Data Analysis

In order to test the hypothesized model (see Figure 1), Structural Equation Modeling analysis was conducted using AMOS 26 version. Overall goodness of fit was assessed by a variety of indices: goodness of fit index (GFI, recommended result should be higher than .90); adjusted goodness of fit index (AGFI, recommended result should be higher than .85); normed fit index (NFI, recommended result should be higher than .90); Tucker–Lewis index (TLI, recommended result should be higher than .95); comparative fit index (CFI, recommended result should be higher than .95); incremental fit index (IFI, recommended result should be higher than .95); and the Root-mean-square-Error-of-Approximation (RMSEA, recommended result should be lower than .08) ( for more information about the goodness of fit cutoffs, see: Bentler, 1990; Hu & Bentler, 1995; Marsh et al., 1988).

## Results

Table 1 presents descriptive statistics and correlations between the model variables. As indicated in the table, all the model variables are significantly associated with one another. Therefore, data were tested for multicollinearity. The analysis revealed variance inflation factor (VIF) estimates ranging between 1.192 and 1.054, indicating low multicollinearity (Thompson, Kim, et al., 2017). Then a Structural Equation Modeling analysis was conducted. Table 2 summarizes the standardizes factor loadings and regression weights of the model’s variables. As indicated in the table, all regression weights are statistically significant. In addition, the fit indices indicate a very good fit of the theoretical model with the data (*GFI* = 0.999, *AGFI* = 0.980, *NFI* = 0.997, *TLI* = 1.000, *CF I* = 1.000, *IFI* = 1.000, *RMSEA* = 0.004), confirming the mediation effect of exposure to information, fear of contagion, and symptoms of hypochondriasis on the association between death anxiety and adjustment disorder symptoms.

**Table 1.** Descriptive Statistics and Correlations Among the Model’s Variables.

Variable	Mean/%	S.D.	Death anxiety	Hypochondriasis	Adjustment disorder	Exposure to information	Fear of contagion
Death anxiety	1.786	0.425	1	0.447**	0.343**	0.180**	0.347**
Hypochondriasis	1.864	0.669		1	0.485**	0.203**	0.393**
Adjustment disorder	7.268	3.045			1	0.338**	0.606**
Exposure to information						1	0.324**
Not at all	0.7						
Once in every few days	10.9						
Every other day	10.9						
Every single day	37.8						
Several times a day	37.8						
Fear of contagion							1
Not at all	17.2						
A little	40.4						
Sometimes	23.8						
A lot	13.6						
Very much	5.0						

**p < .001.

**Table 2.** Standardized Factor Loadings and Regression Weights.

	Standardized factor loading	Regression weights
Exposure to Information <--- Death anxiety	0.180	0.422*
Fear of contagion <--- Death anxiety	0.347	0.882**
Hypochondriasis <--- Death anxiety	0.447	0.703**
Adjustment disorder <--- Exposure to Information	0.134	0.409*
Adjustment disorder <--- Fear of contagion	0.453	1.277**
Adjustment disorder <--- Hypochondriasis	0.280	1.273**

*p < .01, **p < .001.

Consistent with the study’s hypotheses, positive associations were found between death anxiety and all three mediators as well as between all three mediators and adjustment disorder symptoms, indicating that higher levels of death anxiety symptoms increase the extent to which participants are exposed to information regarding the spread of the pandemic, as well as the fear of contagion and symptoms of hypochondriasis, which in turn increase symptoms of adjustment disorder.

## Discussion

The present study aimed to explore mechanisms underlying the association between death anxiety and symptoms of adjustment disorder in the face of the outbreak of COVID-19 pandemic. The results of our study confirmed the mediation model, revealing that higher levels of death anxiety increase the extent to which participants are exposed to information regarding the COVID-19 pandemic, as well as the fear of contagion and symptoms of hypochondriasis, which all in turn increase symptoms of adjustment disorder. Indeed, studies have demonstrated that heightened stress responses during and in the immediate aftermath of a threatening event are associated with adverse physical and mental health outcomes over time (Garfin et al., 2020), including adjustment disorder (Maercker et al., 2013).

These findings may be understood through the prism of TMT (Greenberg et al.,1997), which postulates that there are several coping mechanisms which aim at warding off death-related cognitions and thoughts and enable everyday functioning (Greenberg et al., 1997). Regarding exposure to information, previous studies conducted through the prism of TMT found that feeling powerful, whether by acquiring more knowledge or through other means, reduces anxiety when mortality is salient and may serve as a source for creating meaning for warding off death-related thoughts (Belmi & Pfeffer, 2016). Generally, anxious individuals often engage in information-seeking activities in order to reduce levels of anxiety. However, they are not always capable of dealing with the content or the amount of information they receive (Kuang & Wilson, 2020). Indeed, our research findings demonstrate that when mortality is salient due to a life-threatening pandemic, individuals attempt at feeling powerful and gain control through exposure to information regarding the life-threatening situation. Nevertheless, as the very information they are exposed to is threatening by itself, due to its death-related content and high frequency, anxiety levels increase and individuals show symptoms of adjustment disorder. This finding is in line with previous research linking exposure to information through the media and anxiety and stress-related disorders (Cohen et al., 2006; Ring et al., 2018; Tausczik et al., 2012). Regarding the second mediator, fear of contagion, our research findings are consistent with TMT (Greenberg et al., 1997) which emphasizes the connection between increased death awareness and subsequent anxiety. Our research finding demonstrate how when death awareness is increased due to a worldwide life-threatening pandemic outbreak, subsequent anxiety may arise in the form of fear of contagion. This fear may act as an excessive worry which leads to adjustment disorder symptoms (Maercker et al., 2013).

Subsequent anxiety to death awareness may also arise in the form of hypochondriasis symptoms, which was confirmed as the third mediator between death anxiety and adjustment disorder. Our research findings demonstrate that individuals who experience heightened levels of death anxiety in the face the COVID-19 outbreak may become increasingly preoccupied with their health, thereby experiencing hypochondriasis symptoms. Such symptoms may act as recurrent and distressing thoughts about the stressor or constant rumination about its implications which reduces individuals' ability to engage in meaningful activities, and thus, lead to adjustment disorder.

In line with TMT, a worldwide pandemic outbreak such as COVID-19, may create a disruption in one’s anxiety buffering mechanisms, which normally provide protection against anxiety in general and death anxiety in particular. Such disruption may increase individuals’ awareness of their own mortality, as well as a perceived lack of control over the threatening situation, therefore creating heightened levels of death anxiety. Our findings demonstrate how individuals that experience heightened levels of death anxiety, become increasingly preoccupied with the COVID-19 outbreak and present symptoms of maladjustment to the stressful situation. Due to the disruption in their anxiety buffering mechanisms, participants’ impaired ability to suppress thoughts related to illness or death, leads to an overall experience of vulnerability to threat. This vulnerability results in a frequent exposure to information regarding the COVID-19 outbreak (i.e., preoccupation with the stressor), fear of contagion (i.e., excessive worry), and hypochondriasis symptoms (i.e., recurrent and distressing thoughts about the stressor or constant rumination about its implications). All of these led to adjustment disorder symptoms associated with loss of interest in work, social life, caring for others, leisure activities resulting in impairment in social or occupational functioning (restriction of social network, conflicts in family, absenteeism and so on) (Maercker et al., 2013).

Our research should be interpreted in light of several limitations. First, it should be noted that this study was based on self-reports and was collected using a cross-sectional design. Moreover, most of the sample consisted of women, and future studies may wish to examine the issue of gender, especially in light of previous studies which found that across disasters, risk factors for psychological consequences include the severity of the disaster as well as personal characteristics (Bromet et al., 2011; Shindo et al., 2012). Finally, it should be noted that our research was conducted during a time when there were less than 220 confirmed cases of COVID-19 in Israel and no deaths. Future studies thus should explore our findings at times when threat is more salient and concrete.

Despite these limitations, the current study is, to our knowledge, one of very few research efforts which examined the underlaying mechanisms that trigger adjustment disorder symptoms in the wake of death anxiety. Specifically, this study is the very first to reveal that death anxiety symptoms increase the extent to which individuals are exposed to information regarding the spread of the pandemic, as well as the fear of contagion and symptoms of hypochondriasis, which all in turn increase symptoms of adjustment disorder.

Finally, as stated above, our research was conducted in Israel, a terror-stricken country, where death anxiety levels are interrelated with the ongoing national security threat (Mahat-Shamir et al., 2018). Moreover, at the time this research was conducted there were less than 220 confirmed cases of COVID-19 and no deaths. Thus, the threat was less tangible. Nevertheless, our findings indicate that the world is indeed a “global village”, as a threat must not be salient in the immediate reality to lead to higher levels of death anxiety which in turn increase the extent to which participants are exposed to information regarding the COVID-19 pandemic, as well as the fear of contagion and symptoms of hypochondriasis, which all in turn increase symptoms of adjustment disorder.

In light of our findings, health care policy makers are encouraged to take the publics' risk for developing adjustment disorder under consideration when addressing the possible negative consequences of a life-threatening pandemic outbreak. Moreover, as our research was conducted at the very beginning of the pandemic outbreak in Israel it indicates on individuals' reactions in times when threat is salient mainly through exposure to information provided through the media, social networks and interactions. Similar evidence for such an effect were found in previous research as for example, the incidence of Ebola in the United States was quite low during the 2014 outbreak, but a nationally representative sample of U.S. residents (*N* = 3,447) showed that heightened media exposure to Ebola-related stories was associated with increased distress, worry, and impaired functioning (Thompson, Garfin, et al., 2017). Thus, our findings demonstrate how during an ongoing threat from a novel disease outbreak such as COVID-19, timely updates from trusted sources about the relative risk of contracting the novel disease versus a more common one is critical. Without them, public fears may escalate, fuel rumors, and provoke stress responses (Garfin et al., 2020) that may ultimately lead to the development of adjustment disorder symptoms. Therefore, it is important for media policymakers to take into account not only rating considerations when mass covering a pandemic outbreak but also to consider possible consequences on the general population’s well-being. Lastly, our results may provide a better understanding of the importance of anxiety buffering mechanisms which are aimed at warding off the awareness of personal mortality of individuals who are subjected to a pandemic outbreak, and to how a disruption in these defense mechanisms may lead to the development of adjustment disorder symptoms. Health care professionals are recommended on taking an active stance in asking for patients’ narratives regarding their death anxiety (using direct questions or by using a death anxiety questionnaire), and accordingly should help clients gain more perceived control over the stressful situation by offering psychological intervention (such as existential or cognitive-behavioral therapies) that can successfully treat death anxiety (Iverach et al., 2014) and thus, reduce individuals' risk for developing adjustment disorder.
